# Trajectories across the healthy adult lifespan on sense of direction, spatial anxiety, and attitude in exploring places

**DOI:** 10.3389/fpsyg.2023.1240873

**Published:** 2023-08-08

**Authors:** Veronica Muffato, Laura Miola, Francesca Pazzaglia, Chiara Meneghetti

**Affiliations:** ^1^Department of General Psychology, University of Padova, Padova, Italy; ^2^Inter-University Research Center in Environmental Psychology (CIRPA), Rome, Italy

**Keywords:** sense of direction, spatial anxiety, attitude in exploring, exploration tendency, aging, trajectory, gender, education

## Abstract

**Introduction:**

Self-evaluations about orientation and navigation in the environment contribute to individual differences in spatial cognition. Evidence suggests that they may change, even slightly, with the progression of adulthood. It is necessary to improve the framing of environment-related subjective self-evaluations in adulthood and aging by examining how they change and the factors related to them. Therefore, this study aimed to examine the developmental trajectories of sense of direction, spatial anxiety, and attitude in exploring place across the adult lifespan while also considering gender and education.

**Materials and methods:**

A sample of 1,946 participants (1,068 women), aged 18–87 years, completed the sense of direction and spatial representation, spatial anxiety, and attitude in exploring scales.

**Results:**

The regression models showed a linear increase in sense of direction with age, stable spatial anxiety until age 66 years when anxiety began increasing, and a stable attitude in exploring with a deflection by age 71 years. Gender played a role in all three types of self-evaluations, with men reporting higher ratings in sense of direction and attitude toward exploring (especially in older men), and lower levels of spatial anxiety than women did. Education also played a role, with higher education years associated with lower ratings in spatial anxiety and a higher sense of direction, nullifying gender differences in the latter.

**Discussion:**

These results offer, in the spatial cognition framework, a better understanding of how specific environment-related self-evaluations develop with age and related factors, such as education. This underscores the importance of enhancing them, particularly in women and older adults.

## 1. Introduction

The ability to navigate and orient oneself in the environment is essential for successfully reaching destinations (e.g., wayfinding) and avoiding getting lost in daily life. This is relevant for the general population but especially for older adults because their ability to move and reach places independently is a crucial indicator of healthy aging and a fundamental pillar of international policies. Indeed, with increasing age, it is widely known that navigation abilities decline in healthy older adults (Head and Isom, 2010; Klencklen et al., 2012; Coutrot et al., 2018; van der Ham et al., 2020); however, it cannot be overgeneralized due to several factors ranging from external (e.g., environment characteristics, source of learning, and type of knowledge assessed) to internal ones (e.g., familiarity, Lopez et al., 2021; cognitive abilities and inclinations, Meneghetti et al., 2022). The latter consists of inclinations related to everyday navigation ability that can be expressed through self-evaluations capturing the lived experience of individuals within space (Heward et al., 2021). These self-evaluations have a role in actual navigation performance; indeed, together with spatial abilities (such as visuospatial working memory and mental rotation), they are related to navigation and environment learning accuracy in both young (e.g., Meneghetti et al., 2021) and older adults (e.g., Muffato et al., 2021, 2022a,b).

Among the spatial self-evaluations, most studies across the lifespan have considered the sense of direction, that is, the ability to locate and orient oneself with respect to environmental space (Hegarty et al., 2002), and spatial anxiety, that is, the degree of anxiety and fear of getting lost during navigation (Lawton, 1994). More recently, research on older adults has also considered the attitude in exploring, i.e., the tendency to explore and the pleasure derived from doing so. All these self-evaluations can be grouped under the name of wayfinding inclinations, that is, the individual preferences and attitudes related to wayfinding (Meneghetti et al., 2021). In both young adults and across the entire lifespan, these inclinations can be considered a single factor (Meneghetti et al., 2014, 2021) that contributes to defining one's spatial profile (He and Hegarty, 2020). Of note, and what inspired our study's research issues, although navigation and spatial learning accuracy seem to decrease in older adults, spatial self-evaluations show some changes across age, with a slight increase or decrease depending on the type of self-evaluations (see following paragraphs) and these self-evaluations still positively relate with environment learning accuracy (Meneghetti et al., 2014). Therefore, the potential role of self-evaluations in environmental knowledge acquisition across the lifespan highlights their relevance as individual factors to consider. There is a need to gather evidence on how spatial self-evaluations progress and develop with age. This study's main aim is to examine the wayfinding attitudes—regarding a sense of direction, spatial anxiety, and attitude in exploring—across the adult lifespan, analyzing the developmental trajectories from 20 to 80 years old.

The evidence available on these self-evaluations in young vs. older adults' studies and other related factors (e.g., gender, relevant in spatial cognition; Nazareth et al., 2019) is now presented.

Concerning the sense of direction, there is evidence in favor of its stability or change with an increase in rating. In fact, research has found that it is stable across the lifespan (when considered at the continuous level; Borella et al., 2014; Taillade et al., 2016; Muffato et al., 2020a,b, 2022a,b; or by young vs. older adults comparison) or that it increases with age (Condon et al., 2015). Moreover, when a single question on navigation ability (bad to very good) is asked, which partly resembles a sense of direction, as made in the Sea Hero Quest project (Cheng et al., 2022; Spiers et al., 2023; Walkowiak et al., 2023), the rating increases with age. Cheng et al. (2022) used machine learning to show that gender, daily commute time, and education level were the main predictors of self-reported navigation ability in a large number of participants (770,000 participants aged 19–70 years). People who commuted longer had tertiary education, and men rated themselves as better navigators. Concerning the role of gender, the study by Walkowiak et al. (2023) found that men referred to a higher self-estimation of their navigation ability than women did. Specifically, older men (aged 60–70 years) self-rated their navigating skills more favorably (“very good”) than younger men did (aged 19–29 years), while older women's self-estimation seems to be similar to the ones of younger women. Moreover, participants' self-estimation positively related to navigation performance, measured by the distance traveled to find a target using a videogame (a shorter distance is better). The results showed that navigation performance was predicted by age (decline as aging), gender, home environment (the structure of the living place), self-estimation on navigation ability, education, and commute time. Furthermore, the relation between navigation performance and self-rated navigation ability is linked to cultural aspects, with greater overconfidence in men observed in nations with male-stereotyped roles. This study confirmed the association between subjective and objective navigation ability, which previous studies have also observed in both young adults (Hegarty et al., 2002; Ishikawa and Montello, 2006) and older adults (Meneghetti et al., 2014; van der Ham et al., 2020). The ratings increase with aging (even other evidence shows stability; Borella et al., 2014; Muffato et al., 2022a,b) and to other factors such as gender and education (Cheng et al., 2022; Walkowiak et al., 2023).

Spatial anxiety is another well-proven factor (Lawton, 1994) that negatively affects navigation and environment learning across the lifespan (Meneghetti et al., 2014; Fornara et al., 2019). Regarding older adults and age, evidence is mixed considering some studies have found no correlation between age and spatial anxiety (Carbone et al., 2019) while others found an increase in spatial anxiety with increasing age (Borella et al., 2014; van der Ham et al., 2020, 2021) and lower spatial anxiety at 30–39 years old than 18–29 years old (van der Ham et al., 2021) which is then higher after 70 years old (Borella et al., 2014). Gender differences (with higher levels of spatial anxiety observed in women compared to men) have been found in younger adults (Lawton, 1994; Alvarez-Vargas et al., 2020) with some evidence in older adults too (van der Ham et al., 2021). The role of spatial anxiety in relation to other factors is less explored in aging, and again, the education level can relate to this self-evaluation (Muffato et al., 2020a,b, 2022a,b). Furthermore, it has been well proven that spatial anxiety negatively relates to environmental performance in young adults (Lawton, 1994) with evidence also in older adults (Muffato and De Beni, 2020; Muffato et al., 2022a,b), or at least at a trend level (van der Ham et al., 2020).

Attitude in exploring is another wayfinding inclination that catches individuals' tendencies of explorer mobility (Pappalardo et al., 2015). It relates to functional spatial beliefs, such as to succeed in a spatial task—self-efficacy—and to improve navigation ability (He and Hegarty, 2020; Miola et al., 2023). Attitude in exploring seems comparable in younger and older adults (Muffato et al., 2020a,b) or slightly decreases across age (Meneghetti et al., 2014; Muffato et al., 2022a). When examined, gender differences emerged in favor of men (Miola et al., 2023) or not in favor of men (Pazzaglia et al., 2018) in young adults, while it has been scarcely examined in older adults. The attitude in exploring was shown to have a positive role in navigation and environment learning in young adults (Mitolo et al., 2015; Pazzaglia and Meneghetti, 2017) with evidence also in older adults (Muffato and De Beni, 2020; Muffato et al., 2020a,b). Pleasure in exploring supports the environment learning in older adults, especially in feasible tasks (e.g., route repetition after route learning; Muffato and De Beni, 2020).

Overall, this overview showed (even with some exceptions) that wayfinding inclinations exhibit slight fluctuations with age, with an increase observed across age (for the sense of direction and spatial anxiety) with gender differences (higher sense of direction/navigation ability and lower spatial anxiety in men compared to women). Finally, other factors, such as education, can have a role. Less evidence is available for attitude in exploring, which appears to be stable or slightly decreasing across the lifespan, with little evidence of other associated factors.

This study aims to examine the trajectories of self-reported sense of direction, spatial anxiety, and attitude toward orientation tasks in individuals aged 18 to over 80 years old, determining whether these trajectories are linear, increasing/stable/decreasing, or have breakpoints. The potential role of gender and years of education in self-reported wayfinding inclinations is conjointly considered. The analysis of trajectory is an interesting approach to examine the development of spatial skills with age, which is frequently used in the development domain from infancy to adolescence (Doerr et al., 2021; Hodgkiss et al., 2021) with some evidence in the elderly (Karlsson et al., 2015). We hypothesize that aging may increase a sense of direction (Cheng et al., 2022) and spatial anxiety (van der Ham et al., 2021) and decrease attitudes toward exploring (Muffato et al., 2022a,b). However, analyzing trajectories enables us to detect whether there is a specific age range where a significant change occurs. This potential breaking point may correspond, albeit with the specificity of each self-evaluation, to the reduction in the frequency of going out and the ability to reach destinations, as observed in individuals aged 65–84 years compared to younger people (Muffato et al., 2022b). Conversely, it may also occur around the age of 60 years (Kirasic, 2000; van der Ham et al., 2020), or even earlier in adulthood (Yu et al., 2021), when a certain level of decline in environment learning performance begins. We expect gender differences (favoring men) in the sense of direction and spatial anxiety (Lawton, 1994), and we will explore the gender effect on exploration tendency. Years of education can have a role as well (Walkowiak et al., 2023). Gender and years of education may have different effects as a function of aging (van der Ham et al., 2020; and spatial anxiety; Walkowiak et al., 2023), and we might expect interactions with age.

## 2. Method

### 2.1. Participants

The study involved 1,946 participants (1,068 women) aged 18–87 years recruited through word of mouth. Inclusion criteria were no history of psychiatric or neurological diseases, or diseases capable of causing cognitive, visual, auditory, and/or motor impairments (Crook et al., 1986); and normal cognitive functioning as assessed by a score of at least 22 in the Montreal Cognitive Assessing (MoCA; Nasreddine et al., 2005; see Bosco et al., 2017, 2020; for the cutoff of the Italian normative sample) for participants aged 60 years or older. A power analysis using the “pwr” library in R for regression models with one breakpoint indicated a sample size of 856 participants before and after the breakpoint (1,712 total) to obtain a power of 0.80, an effect size of 0.10 (small effect size based on previous research; Borella et al., 2014; Condon et al., 2015), and a *p*-value of 0.001. Table 1 shows the demographic characteristics (gender, age, and years of education) of the sample for age decades (this categorization was used for descriptive purposes given age was treated as a continuous variable in the analyses). Younger groups had more years of education than older people did, *F*_(6, 1, 939)_ = 60.06, *p* < 0.001, η^2^*p* = 0.16^1^, as reflecting typical schooling level differences in the Italian population due to the cohort effect (ISTAT, 2011). The local ethical committee approved the study, and all participants were informed of its purpose.

**Table 1:** Demographic characteristics of the sample.

	**18–29 years old**		**30–39 years old**		**40–49 years old**		**50–59 years old**		**60–69 years old**		**70–79 years old**		**80–89 years old**	
	**Women**	**Men**	**Women**	**Men**	**Women**	**Men**	**Women**	**Men**	**Women**	**Men**	**Women**	**Men**	**Women**	**Men**
*N* %	505^+^ 59.7%	341^+^ 40.3%	84 52.8%	75 47.2%	56 52.3%	51 47.7%	55 45.8%	65 54.2%	207 52.9%	184 47.1%	140 50.5%	137 49.5%	21 45.7%	25 54.3%
*M* Age	22.54	23.73	34.14	33.84	44.48	44.1	54.18	54.74	64.72	65.01	73.74	73.98	82.14	82.56
(SD)	2.55	2.51	2.84	3.07	3.18	3.09	2.94	2.88	2.74	2.67	2.71	2.87	1.88	2.27
*M* years of education	14.60	14.76	16.06	14.73	14.5	14.2	12.64	12.74	12.31	12.53	11.59	12.16	10.52	10.6
(SD)	2.11	2.43	2.47	3.24	3.48	3.32	2.84	3.54	3.65	3.58	3.94	4.12	4.97	4.02
*M* MoCA	/	/	/	/	/	/	/	/	27.41	27.04	26.77	26.71	25.38	26.12
(SD)	/	/	/	/	/	/	/	/	1.70	1.79	1.79	2.18	2.13	2.19
*M* SDSR	34.27	38.73	36.26	39.48	34.71	40.35	34.73	39.85	35.24	41.92	36.19	42.82	37.76	39.2
(SD)	7.03	8.19	6.98	7.49	8.46	6.93	8.24	8.15	9.32	7.52	7.74	8.3	7.97	6.83
*z*-scores *M* SDSR	−0.37	0.16	−0.13	0.25	−0.32	0.36	−0.32	0.30	−0.26	0.55	−0.14	0.65	0.05	0.22
(SD)	0.84	0.98	0.84	0.90	1.01	0.83	0.99	0.98	1.12	0.90	0.93	1.00	0.96	0.82
*M* SAS	23.96	19.55	22.69	19.01	23.48	19.63	23.36	21.02	23.76	19.15	23.91	21.66	27.29	23.4
(SD)	6.78	6.68	6.11	6.54	6.58	7.38	8.05	6.22	7.54	6.53	7.33	7.14	7.03	7.09
*z*-scores *M* SAS	0.26	−0.35	0.08	−0.43	0.19	−0.34	0.18	−0.15	0.23	−0.41	0.25	−0.06	0.72	0.18
(SD)	0.94	0.93	0.85	0.91	0.92	1.03	1.12	0.87	1.05	0.91	1.02	0.99	0.98	0.99
*M* AtOT	36.41	39.54	36.26	38.89	36.29	38.16	35.93	39.03	35.1	41.73	33.49	40.98	28.90	37.4
(SD)	7.01	7.66	6.31	7.24	6.88	6.72	8.68	6.92	8.95	6.76	8.68	7.61	9.18	6.71
*z*-scores *M* AtOT	−0.15	0.25	−0.16	0.17	−0.16	0.07	−0.21	0.18	−0.31	0.52	−0.51	0.43	−1.09	−0.02
(SD)	0.88	0.96	0.80	0.91	0.87	0.85	1.09	0.87	1.13	0.85	1.09	0.96	1.16	0.85

SDSR, sense of direction and spatial representation scale (score range 13–65); SAS, spatial anxiety scale (score range 8–48); AtOT, attitude toward orientation tasks (score range 10–60).

+ There is a significant difference in the proportion of women and men across the age groups ($\chi_{\left(6\right)}^{2}$ = 16.710,· Cramer's V = 0.09,·p = 0.010), driven by a different proportion of men and women in the 18- to 29-year-old group (p < 0.001), while the proportions in the other groups did not differ.

### 2.2. Materials

The scales were taken from an Italian battery (De Beni et al., 2014). Participants' responses were assessed using a Likert scale (1 “not at all” to 5/6 “very much”), and a single score was calculated by summing all item ratings.

#### 2.2.1. Sense of direction and spatial representation scale

This scale measures (see also Pazzaglia and Meneghetti, 2017) the individual's sense of direction survey preference (“I'm a person with a good sense of direction”), the usage of cardinal points, and the preferences for the route-landmark-based mode (13 items; Likert scale 1–5; max score 65; Cronbach's α normative and current samples: 0.77, 0.81).

#### 2.2.2. Spatial anxiety scale

This scale measures the degree of anxiety experienced (see also Lawton, 1994) in wayfinding situations (e.g., “Going to an appointment in an unfamiliar part of the city”; eight items; Likert scale 1–6; max score 48; Cronbach's α normative and current samples: 0.87, 0.87).

#### 2.2.3. Attitudes toward Orientation Tasks Scale

This scale measures the individual's attitudes in exploring (e.g., “I like to find new ways in which to reach familiar places”; 10 items, five negatives, reversing the negative items for the total score; Likert scale 1–6; max score 60; Cronbach's α normative and current samples: 0.81, 0.75).

### 2.3. Procedure

Participants in a quiet room (520 online^2^) completed a questionnaire on age, gender, years of education, the MoCA (people over 60 years old), the sense of direction and spatial representation scale (SDSR), attitudes toward orientation tasks scale (AtOT), and spatial anxiety scale (SAS) questionnaires (15 min) in a balanced order, during an individual session guided by an experimenter.^3^

## 3. Results

The data analyses were conducted with the R software (version. 4.2.2). First, we computed descriptive statistics (see Table 1) and correlations between measures (see Supplementary Table S1).

### 3.1. Analyses of the developmental trajectories

Linear regression and non-linear segmented trajectory (using the “segmented” library of R; Muggeo, 2008) models were tested for each wayfinding inclination measure (SDSR, SAS, and AtOT). The following models were compared: (a) the null model; (b) the additional model with age, gender, and years of education as predictors (given the effect of age, gender, and—when examined—education level; Cheng et al., 2022; Walkowiak et al., 2023); (c) the interaction model with interactions between age, gender, and years of education; and (d) the segmented model considering that age may have breakpoints (run on the additive or interaction model based on model selection). For model selection, we chose the best fitting model based on the Akaike Information Criterion (AIC, lower is better; Akaike, 1974) and a significant *p*-value between models (*p* < 0.05). Table 2 shows the model selection with the final best fitting models: the interaction model for a sense of direction with no breakpoint, the segmented model of the additive linear model for spatial anxiety, and the segmented model of the interaction linear model for attitude toward orientation tasks.

**Table 2:** Model selection based on AIC.

	**Sense of direction and spatial representation**	**Spatial anxiety**	**Attitude toward orientation tasks**
AIC null model	13,779.33	13,199.02	13,587.88
AIC linear additive model	13,541.19^***^	130,48.25^***^	13,397.39^***^
AIC linear interaction model	**13,532.90** ^**^	13,048.12	**13,373.25** ^***^
AIC segmented model	13,533.22	**13,039.46** ^**^	**13,360.60** ^***^

In bold type, the AIC value corresponds to the best fitting model.

** p < 0.01.

*** p < 0.001.

The segmented models are run on the best fitting linear model (linear additive model for spatial anxiety and linear interaction model for sense of direction and spatial representation, attitude toward orientation tasks).

#### 3.1.1. Sense of direction and spatial representation best fitting model

**Figure 1:**
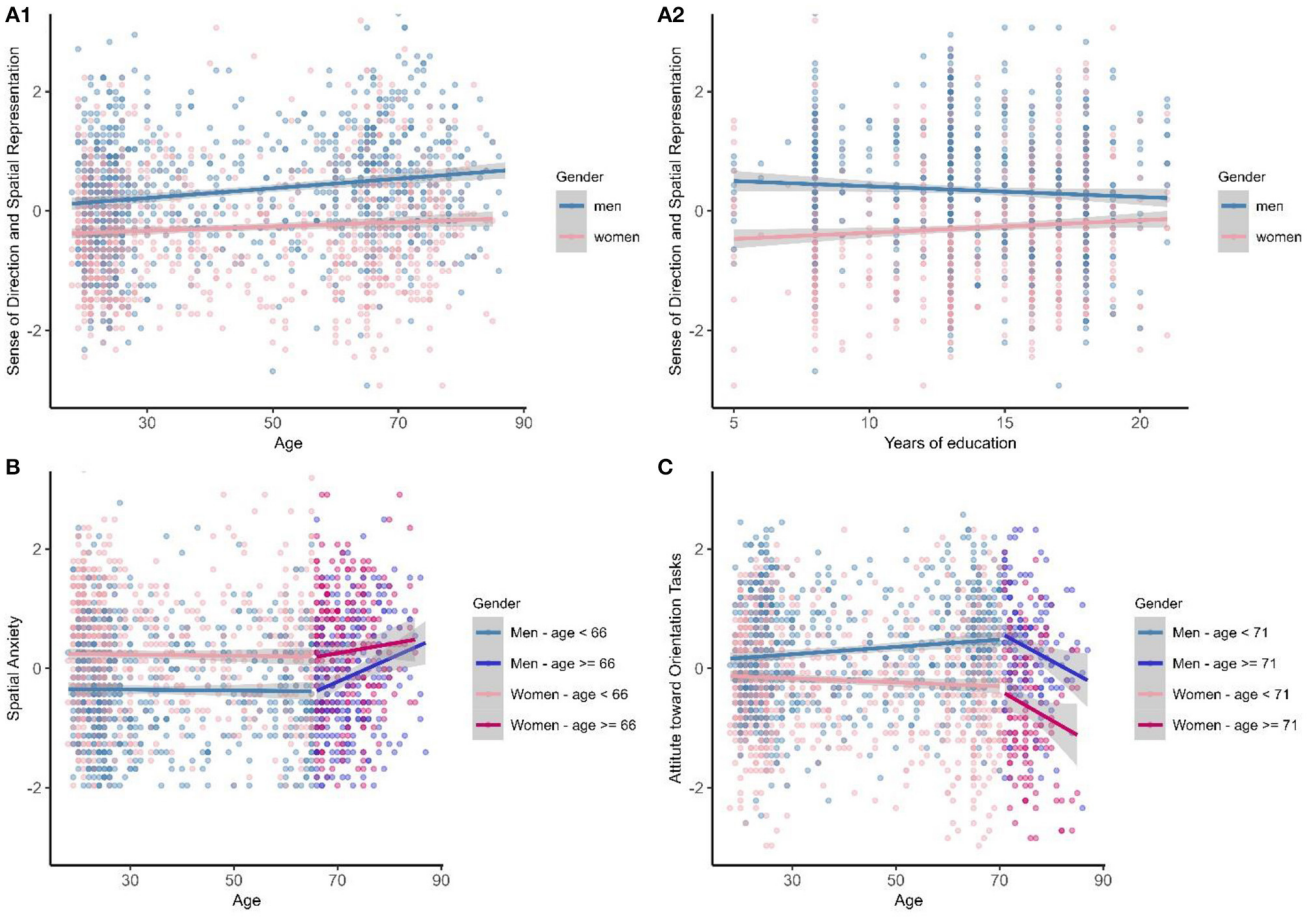
Graphical representations of the three self-evaluation ratings and related variables (standardized scores in ordinate). Sense of direction and spatial representation Scale: **(A1)** Shows the interaction (not significant) between age and gender (it is possible to detect the main effect of age and gender); **(A2)** Shows the (significant) interaction between gender and years of education. Spatial anxiety: **(B)** Shows the interaction (not significant) between age and gender (it is possible to detect the main effects of gender, year of education, and age after the breakpoint, with the breakpoint approximately at age 66 years); attitude toward orientation tasks: **(C)** Shows the (significant) interaction between age and gender (with age after the breakpoint marginally significant; with the breakpoint approximately at age 71 years).

The linear model was the best fitting model (see Table 2; see std. beta, CI, and *p* of the predictors in Supplementary Table S2). Gender, age, years of education, and gender × years of education emerged as significant predictors. The main effects of gender (higher score in men), age (higher score in older adults; see Figure 1A1), and years of education (higher with higher education) were found. The graphical representation of the gender × years of education interaction revealed that among those with the lowest years of education (5), men had higher scores than women did. However, gender differences decreased as years of education increased, and it became null at the highest years of education (20; see Figure 1A2). The overall model had *R*^2^ = 0.13.

#### 3.1.2. Spatial anxiety scale best fitting model

The segmented model (run on the additive model, based on model selection) was the best fitting model (see std. beta, CI, and *p* of the predictors in Supplementary Table S3) and the estimated breakpoint was at 66 years of age [95% CI = (59.58, 72.41)]. After the breakpoint, decreased scores were observed (age after the breakpoint std. beta = 0.12; *p* = 0.009; see Figure 1B). Gender emerged as a significant predictor too (with men reporting lower spatial anxiety than women did; Supplementary Table S3). The overall segmented model had *R*^2^ = 0.08.

#### 3.1.3. Attitude toward orientation tasks best fitting model

The segmented model (run on the interaction model, based on model selection) was the best fitting model, and the estimated breakpoint was at 71 years of age [95% CI = (66.22, 75.16)]. The gender × age interaction emerged as a predictor; young women and men had similar scores that slightly decreased until the breakpoint age, and then, a decrease—although marginally significant—was observed (age after the breakpoint std. beta = −0.14; *p* = 0.058), maintaining gender differences (see Figure 1C; Supplementary Table S4). The overall segmented model had *R*^2^ = 0.12.

## 4. Discussion

This study examines the developmental trajectories of wayfinding inclinations, including sense of direction and spatial representation, spatial anxiety, and attitude in exploring scales, in people aged 18–87 years old. This study offers the opportunity to understand better how self-evaluations change with age and their potential relationship with other factors, such as gender and education. Concerning the predictors, overall, the results showed a distinct age-related pattern in the three self-evaluations: a linear trend for a sense of direction and an age breakpoint for spatial anxiety and attitude in exploring.

Concerning the sense of direction, the regression models showed the role of age, with scores linearly increasing with age. However, the increase observed was modest (as indicated by a small effect, std. beta = 0.12), consistent with previous findings (Condon et al., 2015, *r* = 0.22, *n* = 12,155; Cheng et al., 2022, *r* = 0.02, *n* = 770,000). Furthermore, results showed the main effect of gender, with men having higher self-evaluations than women, as was proven in previous research (Hegarty et al., 2002; Miola et al., 2023), and the main effect of years of education with scores increasing alongside increasing years of schooling (Cheng et al., 2022). The role of years of education was better defined considering the interaction gender × years of education. Specifically, there were gender differences (men having higher scores than women) with the lowest year of education (which were mostly older adults, given that the compulsory education level used to be 5 years). Conversely, there were no gender differences in individuals with higher years of education (20 years). Possibly, people with a lower education level (indication of both low socioeconomic status and older age) and men (Italy—similar to other European Union countries—tend to have masculinity beliefs; Walkowiak et al., 2023) develop more misleading beliefs on navigation ability in comparison to women. Conversely, with increasing schooling levels, the estimation decreased in men and increased in women—becoming similar in both genders—probably making the evaluation less subject to cultural factors and less misleading. It is possible that higher years of education enabled individuals to produce self-evaluations more consistent with their actual performance (less misleading). Although plausible, this is only speculation because actual navigation performance has not been assessed, and more evidence is needed.

Unlike the sense of direction, the spatial anxiety and exploration tendency showed a segmented developmental trajectory, enabling identifying an age range at which ratings change. Concerning spatial anxiety, an age breakpoint can be detected at 66 years old, with spatial anxiety increasing significantly after that age. This is in line with previous evidence (Borella et al., 2014), but the trajectory analysis reveals a new insight: a sharp increase occurring around the age of 66 years. Gender (men reporting lower anxiety than women did) is important too, in line with the literature (Lawton, 1994; van der Ham et al., 2021). It is worth noting that these findings regarding age and gender might be related to the interconnection between spatial anxiety and general trait anxiety (Munoz-Montoya et al., 2019; Mendez-Lopez et al., 2020), even spatial anxiety is considered a distinct individual spatial disposition (Lawton, 1994; He and Hegarty, 2020). Furthermore, a relationship with education was identified (anxiety decreased as years of education increased). This novel finding in the spatial domain is consistent with evidence showing a positive association between education level and wellbeing (including lower levels of anxiety, e.g., Belo et al., 2020). Education is considered a proxy for cognitive reserve (Staekenborg et al., 2020), which individuals can draw upon to cope with environmental demands, thus possibly reducing negative emotions. Education could help to limit the increase in spatial anxiety and potentially mitigate its negative effects on spatial performance. Nevertheless, further evidence is needed to confirm this relationship.

Concerning the attitude in exploring, a breakpoint can be detected around 71 years, even as a tendency. Age appears to be related to gender, as younger men report similar ratings for women, while adult men tend to give higher ratings than women. Around the age of 71 years, both men and women show a decrease in ratings. No effect of education was found. The fact that attitude in exploring is not influenced by education level, that is, it does not depend on socioeconomic status, and instead it would seem to be an individual attitude more related to age and gender, suggests it could be encouraged. Doing so, especially in older women, could become a success factor in actual navigation skills and help older adults maintain confidence and a positive feeling about exploring their environment. Sustaining this positive exploration approach can help counteract the negative effects of spatial anxiety (that increase), as commented above. Studies have shown that the exploration tendency relates to motivational spatial beliefs (such as spatial self-efficacy and incremental view on navigation ability; Miola et al., 2023) and environment learning in aging (Muffato and De Beni, 2020), making further investigation on this wayfinding inclination important.

These results provide insight into the trend of wayfinding inclinations self-ratings over the course of age. Older adults, likely due to accumulated experiences, recognize their orientation abilities (a sense of direction) but experience higher anxiety levels and less positive attitudes. These outcomes may be associated with higher cognitive difficulties experienced by certain older adults. Further research should specifically address this issue.

However, there are limitations to consider. First, this is a cross-sectional study and a longitudinal study would provide more robust evidence about age-related changes in wayfinding inclinations; this should be considered in future studies. Second, the current study only focuses on subjective spatial self-evaluations and it does not include objective measures, such as navigation or environment learning accuracy. While we know that self-evaluations relate to environmental performance in both young and older adults (e.g., Meneghetti et al., 2014), an objective measure of environmental performance would better corroborate the relation between wayfinding inclinations and age, with particular attention when these inclinations change more notably (≥66 years old for spatial anxiety and attitude in exploring). Third, another aspect to note is the high variability of ratings within the same age (as the dots in panels of Figure 1 demonstrate). This indicates large individual differences in wayfinding inclinations (Condon et al., 2015), and additional research is necessary to examine better the factors that contribute to this variability, not only including gender and education (Cheng et al., 2022) but also other factors such as spatial experience and living environment (van der Ham et al., 2020). Other limitations include the absence of a cognitive measure for younger adults to investigate cognitive functioning's impact on self-reports, lacking individuals under 18 years for a complete age spectrum, and the gender imbalance among younger participants. Additionally, the modality of data collection (online vs. in-person) should be considered, as online data may not fully overlap with in-person conditions, despite being practical (Newman et al., 2021).

## 5. Conclusion

This study offers a contribution to enlarging the theoretical framework of spatial cognition concerning self-evaluation of environment orientation and navigation across the adult lifespan. Although there is a linear trend for a sense of direction with age, a shift occurs upon entering aging (~66–71 years old), with an increase in spatial anxiety and a decrease in positive attitudes toward exploration. Other individual factors are at play, such as gender and education. Men have lower levels of spatial anxiety and a higher sense of direction and attitude toward exploring, particularly in older men for the latter. Higher years of education result in a less misleading sense of direction ratings, especially in older men, and decreased spatial anxiety ratings.

Although it is important to consider environmental factors that facilitate orientation, reduce spatial anxiety, and increase pleasure in exploring, it is also crucial to investigate internal factors that can help people enhance and better exploit their self-perception in relation to orientation tasks, particularly as they age.

## Data availability statement

The datasets presented in this study can be found in online repositories. The names of the repository/repositories and accession number(s) can be found at: doi: 10.6084/m9.figshare.23302067.

## Ethics statement

The study was approved by the Ethical Committee of University of Padova (Univocal number: ECB34AD40A1A848DF201D0C38176DEDD). All participants were informed about the purposes of the study and gave their written informed consent in accordance with the Declaration of Helsinki (World Medical Association, 2013). The patients/participants provided their written informed consent to participate in this study.

## Author contributions

CM, VM, LM, and FP contributed to the design, the implementation of research, data collection, the analysis of the results, and the writing of the manuscript. All authors contributed to the article and approved the submitted version.

## Footnotes

^1^After conducting *post hoc* analyses with Bonferroni corrections (significance level at *p* ≤ 0.001), the 18–29, 30–39, and 40–49 age groups did not differ significantly from each other in terms of years of education (0.085 ≤ *p*s ≤ 1). However, these three age groups differed significantly from the 50–59, 60–69, and 70–79 age groups (*p*s ≤ 0.001), which, in turn, did not differ significantly from each other (0.002 ≤ *p*s ≤ 1).

^2^Participants were collected between 2014 and 2021, with 520 participants (341 women) collected online in 2020/2021 due to the COVID-19 pandemic. The same analysis conducted separately for the in-person sample yielded similar results (see Supplementary material).

^3^Participants also completed additional measures such as visuospatial abilities tasks, map learning, and navigation tasks. These measures varied among individuals and were not included in the current study.
