# Supplementary material for: Trajectories across the healthy adult lifespan on sense of direction, spatial anxiety, and attitude in exploring places
Source: Front Psychol. 2023 Aug 8;14:1240873. doi: 10.3389/fpsyg.2023.1240873 (PMC10442537; doi:10.3389/fpsyg.2023.1240873)
Supplement: Supplementary file 1 [file Data_Sheet_1.PDF]

Supplementary Material

Trajectories across healthy adult lifespan on sense of direction, spatial anxiety and attitude in exploring places

Veronica Muffato*, Laura Miola, Francesca Pazzaglia, Chiara Meneghetti*

*** Correspondence:** Chiara Meneghetti or Veronica Muffato, Department of General Psychology, Via Venezia, 8, 35131, Padova, Italy
e-mail: chiara.meneghetti@unipd.it, veronica.muffato@unipd.it

1 Supplementary Figures and Tables

Table S1. Pearson's correlations between variables

	1	2	3	4	5	6
1. Age	-					
2. Gender	**0.09*****	-				
3. Education	**-0.36*****	-0.02	-			
3. SDSR	**0.15*****	**0.32*****	0.00	-		
4. SAS	0.02	**-0.27*****	-0.07**	**-0.37*****	-	
5. AtOT	-0.02	**0.28*****	**0.14*****	**0.54*****	**-0.47*****	-

Note. SDSR = Sense of Direction and Spatial Representation scale; SAS = Spatial Anxiety Scale; AtOT = Attitude toward Orientation Tasks. For r > |07|, $p < 0.001$ (given multiple comparisons, only $p < 0.001$ are considered significant, in bold type).

Table S2. Linear interaction regression model for Sense of Direction and Spatial Representation (SDSR)

	std. Beta	standardized CI	p
Gender	0.63	0.54 – 0.72	**<0.001**
Age	0.12	0.06 – 0.18	**<0.001**
Years of education	0.11	0.04 – 0.18	**0.002**
Gender × Age	0.05	-0.04 – 0.14	0.277
Gender × Years of education	-0.15	-0.25 – -0.05	**0.003**
Age × Years of education	0.01	-0.06 – 0.08	0.769
Gender ×Age × Years of education	0.06	-0.03 – 0.16	0.200

Note. In bold type the significant p values.

Table S3. Segmented additive model for spatial Anxiety Scale (SAS)

	std. Beta	standardized CI	p
Gender	-3.90	-4.51 – -3.28	**<0.001**
Age	-0.01	-0.04 – 0.01	0.261
Years of education	-0.13	-0.23 – -0.03	**0.011**
Age before the breakpoint	-0.03	-0.08 – 0.03	0.332
Age after the breakpoint	0.12	0.03 – 0.20	**0.009**

Note. In bold type the significant p values.

Table S4. Segmented interaction model for Attitude toward Orientation Tasks (AtOT)

	std. Beta	standardized CI	p
Gender	-0.16	-8.98 – 8.66	0.972
Age	-0.04	-0.15 – 0.06	0.395
Years of education	0.29	-0.14 – 0.71	0.185
Age before the breakpoint	-0.02	-0.09 – 0.05	0.543
Age after the breakpoint	-0.14	-0.29 – 0.00	0.058
Gender × Age	0.15	0.01 – 0.30	**0.042**
Gender × Years of education	0.09	-0.52 – 0.69	0.776
Age × Years of education	0.00	-0.00 – 0.01	0.485
Gender ×Age × Years of education	-0.01	-0.02 – 0.00	0.280

Note. In bold type the significant p values.

Given that a portion of the sample was collected online, we conducted additional analyses excluding these participants to assess any potential differences. Please refer to Table S4 for model selection and Tables S5, S6, and S7 for the models for SDSR, SAS, and AtOT, respectively (N = 1426).

Table S4. *Model selection with in-presence sample*

	Sense of Direction and Spatial Representation	Spatial Anxiety	Attitude toward Orientation Tasks
AIC Null model	10169.009	9677.743	10235.12
AIC Linear additive model	9985.577***	9560.982***	10016.16***
AIC Linear interaction model	**9978.159****	9565.819	**10012.18***
AIC Segmented model	9980.959	**9556.813***	**10001.57*****

Table S5. *SDSR results with in-presence sample*

	std. Beta	standardized CI	p
Gender	0.69	0.58 – 0.80	**<0.001**
Age	0.16	0.08 – 0.24	**<0.001**
Years of education	0.15	0.07 – 0.24	**<0.001**

Gender × Age	-0.01	-0.12 – 0.10	0.91
Gender × Years of education	-0.19	-0.30 – -0.07	**0.002**
Age × Years of education	-0.02	-0.10 – 0.06	0.64
Gender ×Age × Years of education	0.12	0.00 – 0.23	**0.056**

Table D. *SAS results with in-presence sample*

	**std. Beta**	**standardized CI**	**p**
Gender	-3.83	-4.55 – -3.11	**<0.001**
Age	0.01	-0.02 – 0.03	0.525
Years of education	-0.10	-0.21 – 0.01	0.066
Age before the breakpoint	0.01	-0.03 – 0.10	0.28
Age after the breakpoint	0.10	0.01 – 0.19	**0.035**

Table E. *AtOT results with in-presence sample*

	**std. Beta**	**standardized CI**	**p**
Gender	12.48	0.63 – 24.32	**0.039**
Age	0.05	-0.09 – 0.20	0.486
Years of education	0.76	0.18 – 1.35	**0.010**
Age before the breakpoint	-0.02	-0.09 – 0.05	0.543
Age after the breakpoint	-0.14	-0.29 – 0.00	0.058
Gender × Age	-0.03	-0.22 – 0.15	0.747
Gender × Years of education	-0.55	-1.32 – 0.23	0.166
Age × Years of education	-0.00	-0.01 – 0.01	0.464
Gender ×Age × Years of education	0.00	-0.01 – 0.02	0.574

frontiers
